# Introduction to subpressure-driven soft deformation method for removing inherent voids in green components manufactured by material extrusion

**DOI:** 10.1016/j.heliyon.2024.e28689

**Published:** 2024-03-28

**Authors:** Taehyeob Im, Heungseok Oh, Byeonghwa Goh, Juyong Kim, Jai-Sung Lee, Joonmyung Choi, Caroline Sunyong Lee

**Affiliations:** a Department of Materials and Chemical Engineering, Hanyang University ERICA, Republic of Korea; b Department of Mechanical Design and Engineering, Hanyang University, 222 Wangsimni-ro, Seongdong-gu, Seoul, 04763, Republic of Korea; c Department of Mechanical Engineering, BK21 FOUR ERICA-ACE Center, Hanyang University, 55 Hanyangdaehak-ro, Sangnok-gu, Ansan, 15588, Republic of Korea; d Reprotech 3DP R&D Center, Yongin-Si, Republic of Korea

**Keywords:** Material extrusion (MEX), Inherent voids, Subpressure-driven soft deformation, All-atom molecular dynamics simulations

## Abstract

This study introduces a post-treatment process, the subpressure-driven soft deformation method, to reduce inherent voids in Material Extrusion (MEX) components. By subjecting printed green components to heat treatment under subpressure, the process enhances viscosity, effectively filling voids formed between deposited tracks. The average porosities of the samples sintered from the green components without and with soft deformation are calculated to be 3.55% and 2.36%, respectively. A comparison of the tensile strengths and fracture surfaces of the sintered samples with and without soft deformation treatment indicated that the sintered samples with soft deformation treatment exhibited narrower standard deviation for the various mechanical properties. Capillary rheometer calculations indicate feedstock viscosity to be between 450.34 and 1018.31 Pa s under subpressure, diminishing inter-track voids without sizeable dimensional changes. Molecular dynamics simulation demonstrates a 3.7-fold increase in bond strength, indicating intertrack voids effectively eliminated. Reduced inter-particle distances facilitate necking, grain growth, and improved sintered density.

## Highlights

• Subpressure-driven soft deformation removes inherent voids formed during printing.
• Soft deformation improves flowability of the green part.
• Soft deformation mechanism explained using all-atom molecular dynamics.
• Inherent voids generated along the extruded line were successfully removed.
• Soft deformation affects sintered sample microstructure/mechanical properties.

## Graphical abstract

**Figure:**
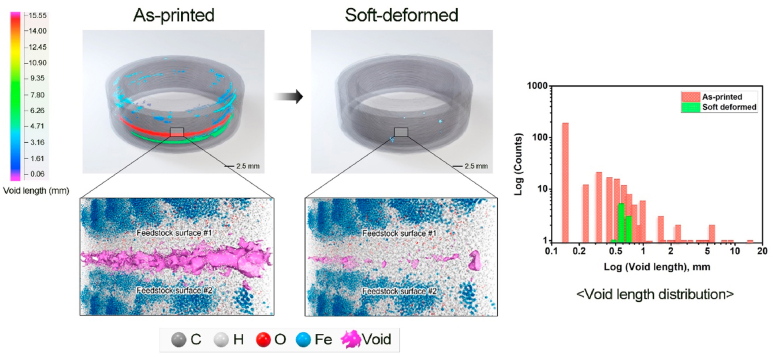

## 1 Introduction

The additive manufacturing of metal components has been investigated extensively to achieve components with the desired properties for various applications. Metal additive manufacturing is being continuously developed for manufacturing components with excellent properties and multifunctionality, expanding its application range, and developing materials and printing technologies suitable for diverse applications [1]. Hardware and process development, material design, and characterization methodologies are closely related and must be understood comprehensively for their future research and development.

Powder bed fusion (PBF), direct energy deposition (DED), and binder jetting (BJT) are the most widely used fabrication methods for metal additive manufacturing. PBF involves selectively scanning a laser onto a printed area after layer deposition on a powder bed. A notable advantage of this method is its ability to print complex shapes at high speed with excellent precision. However, unique defects are formed because of the high-energy source used. This results in mechanical anisotropy, which depends on the orientation of the final printed product with residual stress owing to the high cooling rate. Furthermore, the flowability of the powder determines the properties of the final product; therefore, spherical powders with classified particle sizes must be used [2]. In DED, printing is performed by scanning a high-power laser beam across the surface of a component, thus supplying metal powder to an instantaneously generated melt pool. The products manufactured using DED exhibit excellent mechanical properties. However, they show unique defects owing to their high energy sources, similar to the results of PBF [2]. Moreover, high-energy sources such as lasers and electron beams are expensive. In BJT, a green component is manufactured by adding a binder, such as melted wax, cyanoacrylate glue, or epoxy, to a selected area instead of by scanning a high-energy source after layer deposition on the powder bed. Sintering is required to sinter the green components in which the powder and binder are mixed. Although BJT allows complex-shaped components to be printed at high speed, it requires a spherical powder with a uniform particle size to improve the packing density during layer deposition, thus limiting the shape of the materials to be used [3]. In material extrusion (MEX), various particle sizes ranging from micro- [4] to nano-sized particles [5] can be used, unlike in PBF and BJT, which require spherical powders. In addition, MEX allows numerous types of powders to be used, such as ceramics and composite powders [5] or irregular shape of powders [6]. These characteristics render MEX favorable in the next-generation additive manufacturing. Furthermore, the equipment required for MEX is relatively inexpensive and the feedstocks required can be obtained easily [7]; thus, it is a favorable method. The market share of MEX in metal additive manufacturing has increased to more than 10% [8] and the number of published papers pertaining to metal extrusion has increased [4].

In this study, a new powder-type metal injection molding (MIM) feedstock system is introduced, where powder-based feedstock is directly extruded to print multiple layers with high powder loading such that excellent dimensional stability can be maintained during debinding and sintering. Compared with other MEX methods, the powder-type MIM feedstock system results in a more cost-effective approach owing to its low material cost [9]. However, severe surface roughness [5] and void formation [10] are inevitable between the deposited tracks (intertrack) and layers (interlayer) [11], thus resulting in a weakened structure that degrades the quality of the final printed product [12]. Although the printed green component is surrounded by continuous binders between the deposited tracks, achieving intimate contact between the layers is difficult and inherent voids are generated [10]. Moreover, unlike the fused filament fabrication method, which uses filament-type feedstocks, continuous extrusion with non-filament feedstocks can be challenging and causes voids to be formed more frequently in the green component. These inherent voids formed between the layers cannot be removed even at sintering temperatures of 1000 °C or higher and result in the formation of larger voids due to their expansion at high temperatures. This is a significant problem when using powder-type MEX despite the many advantages listed above.

An innovative method called subpressure-driven soft deformation is proposed herein to reduce the size of inherent voids formed between deposited tracks during printing by applying the viscosity of the green component. Subpressure-driven soft deformation is a method that effectively reduces intertrack voids and improves interlayer adhesion within three-dimensionally printed green component by subjecting them to heat treatment at sufficiently high temperatures to melt the binder under sub-atmospheric pressure. The strategy effectively reduces the microvoids lying between adjacent feedstocks by increasing the fluidity of the viscous binder, which is theoretically demonstrated through all-atom molecular dynamics (MD) simulations. In particular, such a computational approach showed that an increase in binder fluidity can lead not only to a reduction in voids in the interfacial region but also to densification of metal particles contained within the binder bulk. Compared with other methods such as using auxiliary equipment [13], the optimization of feedstocks [12] and process variables [14] for removing these inherent voids has been reported, and the proposed process is simple. Furthermore, subpressure-driven soft deformation can be applied to any other types of green components by regulating the viscosity of the binders to reduce the size of the inherent intertrack voids.

## 2 Material and methods

### 2.1 Feedstock

**Fig. 1:**
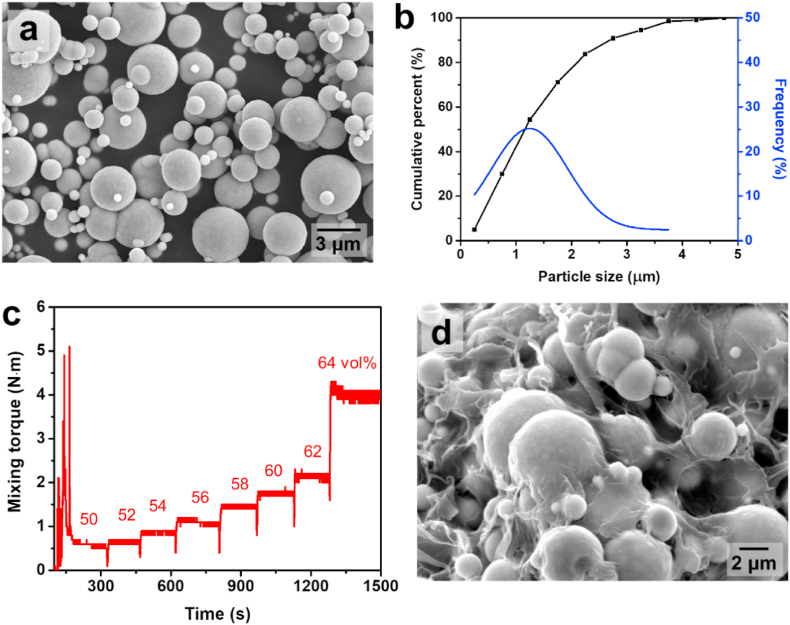
(a) SEM image of single-sized iron powder, (b) particle size distribution of single-sized iron powder, (c) mixing torque for feedstocks ranging from 50 vol% to 64 vol% with respect to time, and (d) SEM image of feedstock after homogeneous mixing using twin screw mixer.

In this study, feedstock was prepared using single-sized pure iron powder (99.9%, Avention Co. Ltd.) and a paraffin wax-based binder (paraffin wax:ethylvinyl alcohol:polyethylene: stearic acid ratio = 57:20:20:3 (wt.%)) (Reprotech Inc.). The iron powder particles were spherical, with a mean particle size of 1.12 μm, as shown in Fig. 1(a) and (b). Fig. 1(c) shows the mixing torque of the feedstock measured using a twin-screw mixer (Plasti-Corder, CW Brabender Instruments, Inc.) to determine the optimal powder-loading volume. The mixer had a capacity of 60 cm^3^, and 65%–75% of the chamber volume was filled with powder and binder [15]. The twin screws were rotated at 60 rpm. The temperature of the chamber was fixed at 145 °C to such that the powder mixed uniformly with the binder. The powder was placed in a mixer before the binder was added. Mixing was performed until the mixing torque reached a specific value. To determine the optimal amount of solid loading in the feedstock for material extrusion, the mixing torque was evaluated by adding 2 vol% powder. Our results showed that the critical loading of feedstock occurred at 62 vol%, which was accompanied by a significant increase in the mixing torque from 62 vol% to 64 vol%. Typically, to achieve smooth three-dimensional (3D) printing via material extrusion, the critical loading amount is reduced from 2 vol% to 5 vol% [4]. Therefore, we selected 60 vol% iron powder to be mixed with the binder as the optimal metal feedstock for material extrusion. The powder and binder were mixed uniformly, as shown in Fig. 1(d).

### 2.2 3D printing

**Fig. 2:**
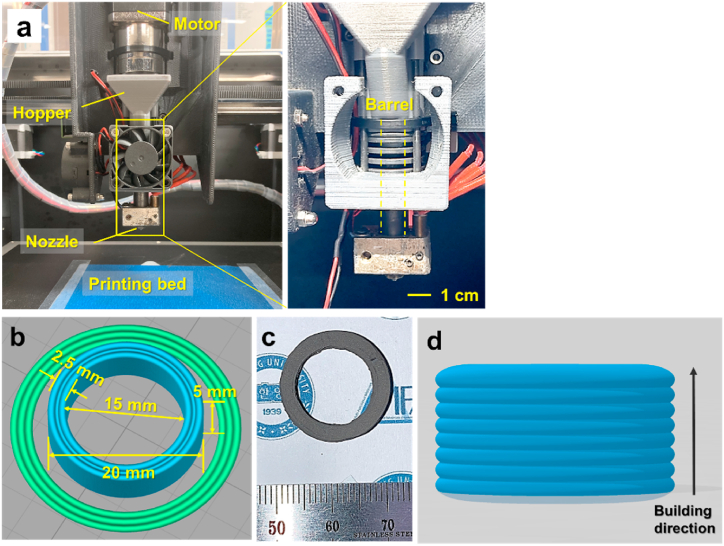
(a) Structure of 3D printer for MEX (yellow box shows magnified view behind cooling fan), (b) 3D model for subpressure-driven soft deformation showing deposited tracks (intertrack), (c) photograph of as-printed green component, and (d) 3D model for layers deposited in building direction. (For interpretation of the references to color in this figure legend, the reader is referred to the Web version of this article.)

A screw-type extrusion 3D printer (CME-300, Reprotech) was used for 3D printing, as shown in Fig. 2(a). The Simplify 3D software (Simplify3D LLC) was used for 3D printing. A nozzle with a diameter of 0.8 mm and a layer thickness of 0.2 mm was used for printing. The 0.8 mm nozzle was adequate to achieve a 0.96-mm-wide deposited track with an extrusion multiplier of 1.5, and the nozzle temperature was set to 150 °C. This setting allowed us to print shapes with a thickness of 2.5 mm. The printing speed was set at 17.5 mm/s. To facilitate the detection of inherent voids between the deposited lines, we printed the material in a toroidal shape, as illustrated in Fig. 2(b). Meanwhile, to minimize void formation in the three-dimensionally printed toroidal shapes, we printed the outer and inner layers first, followed by the center layer between the outer and inner layers to minimize the formation of inherent voids during the printing. The green component printed using this approach, as shown in Fig. 2(c), exhibited dense and void-free regions between the deposited tracks. A 3D model of the typical shapes of layers deposited in the building direction in MEX is shown in Fig. 2(d).

### 2.3 Subpressure-driven soft deformation

The aim of soft deformation is to reduce the size of voids by subjecting a three-dimensionally printed green component composed of MIM feedstock to heat treatment up to its melting temperature. To effectively control the soft deformation process, subpressure control was implemented during the heat treatment of the three-dimensionally printed green component. Subpressure refers to the application of pressures below the atmospheric pressure. Improvements in the bonding strength between adhesive or coating films and substrates by applying subpressure with heat treatment have been reported to improve bonding strength [16] or to remove the void between films [17]. In the MIM feedstock, this control exploits the shear thinning effect, which is a non-Newtonian phenomenon where the viscosity decreases as the shear rate increases [18]. In addition, subpressure was applied during the heat treatment to encourage the flow of fluid in the desired direction to fill the voids formed between the deposited tracks.

The shear rate is proportional to the pressure applied to the molten feedstock; therefore, the viscosity of the fluid decreases with pressure. A capillary rheometer (Instron CEAST SR50) was used to evaluate the highly viscous MIM feedstock. This instrument measures the viscosity of materials based on the shear rate and change in pressure, rendering it ideal for evaluating MIM feedstocks [18]. The barrel diameter of the rheometer used in the experiments was 15 mm, and the length/diameter ratio of the die used was 30/1. In addition, a 70 kN load cell was used. First, the temperature at the onset of fluidity in the feedstock was determined using differential scanning calorimetry (DSC, NETZSCH DSC 204F1 Phoenix). Subsequently, the temperature determined was applied to a capillary rheometer. The viscosity was evaluated using a capillary rheometer at increasing temperature intervals of 5 °C above the melting point of the feedstock, as determined by differential scanning calorimetry (DSC).

**Fig. 3:**
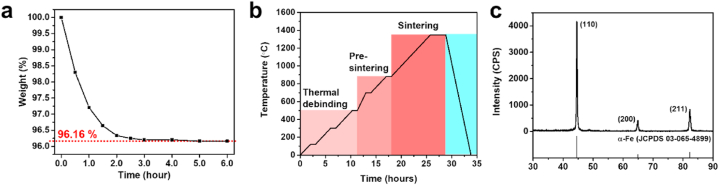
(a) Graph showing weight loss over time during solvent debinding; (b) heating profile of thermal debinding followed by sintering of green components; (c) XRD analysis result of sintered sample. (For interpretation of the references to color in this figure legend, the reader is referred to the Web version of this article.)

For the subpressure-driven soft deformation, the sample was first placed in a tube furnace and heated to the temperature determined via DSC analysis. Subsequently, pressure ranging from 3 to 4 Pa was applied using an oil rotary vacuum pump. The temperature of the furnace was maintained using a thermocouple, which ensured that the temperature did not exceed by 1 °C. The sample was subjected to solvent debinding after the subpressure-driven soft deformation. Solvent debinding was performed by stirring the solution at 40 °C in heptane (Daejung Chemicals). As shown in Fig. 3(a), the sample was stored for at least 6 h until the soluble paraffin wax and stearic acid were completely dissolved. Subsequently, thermal debinding, pre-sintering, and sintering in an Ar/5%H_2_ atmosphere at certain heating rates and temperatures were performed, as shown in Fig. 3(b). Specifically, thermal debinding was performed at 120 °C, 300 °C, and 500 °C for 1 h each under a heating rate of 1 °C/min. Subsequently, pre-sintering was performed at 700 °C and 880 °C for 1 h each at heating rates of 2 and 1 °C/min, respectively. Finally, sintering was conducted at 1350 °C for 3 h at a heating rate of 1 °C/min, whereas XRD analysis was conducted to confirm no indication of oxidation on the sample sintered under a mixed-gas atmosphere (Fig. 3(c)).

### 2.4 Characteristics

Micro-computed tomography (micro-CT, Nikon Metrology XT H 225) was performed to validate the changes in the inherent voids before and after soft deformation. The X-ray spot size was approximately 3 μm. An Archimedes density meter was used to measure the relative densities of green and sintered samples. The cross-sectional images were analyzed via optical microscopy (OM; BX51 M, Olympus) and a field-emission scanning electron microscopy (MIRA3, TESCAN) after they were etched with Nital 2%. The porosities of the sintered samples were analyzed using image analysis software (ImageJ; NIH) by adjusting the contrast in the SEM images, and tensile tests were performed using an MTDI Minos-100 tensile testing machine. To clearly identify the difference in the strength of the tensile specimens based on the soft deformation treatment, the specimens were three-dimensionally printed continuously in directions parallel, vertical, parallel, and vertical to the tensile direction (0°/90°/0°/90°) based on the ASTM E8 standard.

## 3 Viscosity estimation

### 3.1 Viscosity at subpressure

The pressure coefficient (*β*) is a correlated value determined based on various pressures (p) and viscosities (η) when a viscous material is extruded through a rheometer at a uniform temperature (T) and shear stress (σ), as expressed in Eq. (1) [19]: The viscosity at a specific pressure was estimated using Eq. (2) using the pressure coefficient obtained using Eq. (1). The constant c represents the natural logarithm of the viscosity at extremely low pressures.

(1) $$\beta ={\left(\frac{\partial \ln\;\eta }{\partial p}\right)}_{T,\sigma }$$

(2) lnη=β∙p+c

**Fig. 4:**
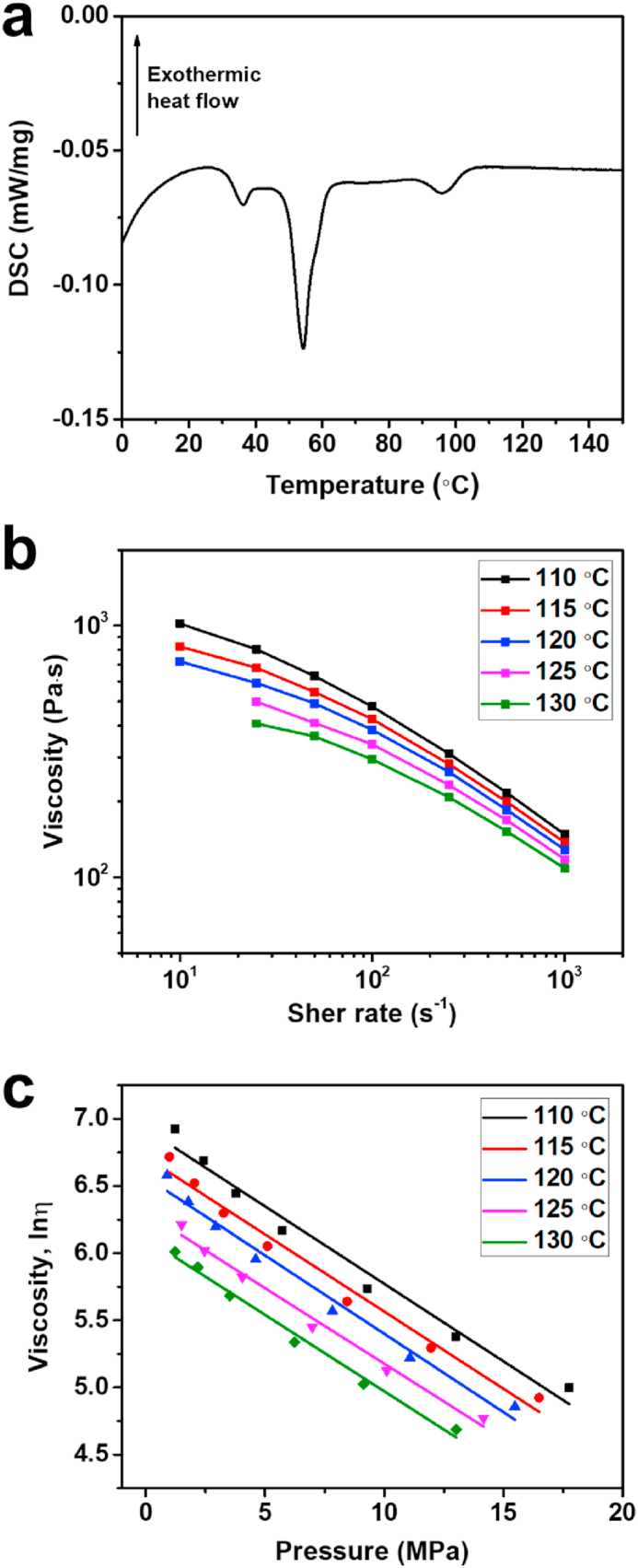
(a) DSC graph of the feedstock; (b) viscosity measurement results based on shear rates at each operating temperature; (c) logarithmic graph of viscosities based on different pressures under the same shear stress.

Fig. 4(a) shows the DSC results of the fabricated feedstock. Three exothermic peaks were observed, which indicated the melting temperature of each polymer in the binder system. The polymer within the feedstock was fully viscous at approximately 105 °C. Capillary rheometer analysis was performed from 110 °C-130 °C at 5 °C intervals based on the DSC results. Fig. 4(b) shows the relationship between viscosity and shear rate. Feedstock analyzed at 125 °C and 130 °C could not be measured at a shear rate of 10 s^−1^ because the binder in the feedstock had melted. Consequently, the viscosity could not be measured. However, we observed that the viscosity decreased significantly at these temperatures during the measurements. Therefore, the value was omitted. The shear-thinning behavior can be inferred from Fig. 4(b). The viscosity decreased as the shear rate increased, and the viscosity of the feedstock ranged from 108 to 1020 Pa s, as measured using a capillary rheometer. In this range, the feedstock melted sufficiently and exhibited flow characteristics. Fig. 4(c) presents a logarithmic graph of the viscosity at varying pressures, showing linear functions correlating the measured viscosity with the applied pressure. The specific values are listed in Table 1. The c values represent the viscosities at extremely low pressures, as expressed in Eq. (2). Subpressures of 3–4 Pa were applied to the printed green component to observe subpressure-driven soft deformation. This pressure was lower than the pressure levels recorded by the capillary rheometer. The viscosities under subpressure were calculated using the c values listed in Table 1. The calculated viscosities differed significantly even within a slight temperature difference of 5 °C. The calculated viscosity ranged from 450.34 to 1018.31 Pa s, which was within the permitted viscosity range of 108–1020 Pa s. This indicates that the binder in the powder forming the 3D green component exhibits flow characteristics even at low pressures.

**Table 1:** Pressure coefficients and viscosites of feedstock under subpressure at varying temperatures.

Temperature (°C)	Pressure coefficient (*β*)	*c* (=ln*η*)	Viscosity (Pa·s)
110	−0.1154	6.9259	1018.31
115	−0.1149	6.7143	824.11
120	−0.1167	6.5668	711.09
125	−0.1125	6.3021	545.72
130	−0.1138	6.1100	450.34

### 3.2 All-atom molecular dynamics (MD) simulations

**Fig. 5:**
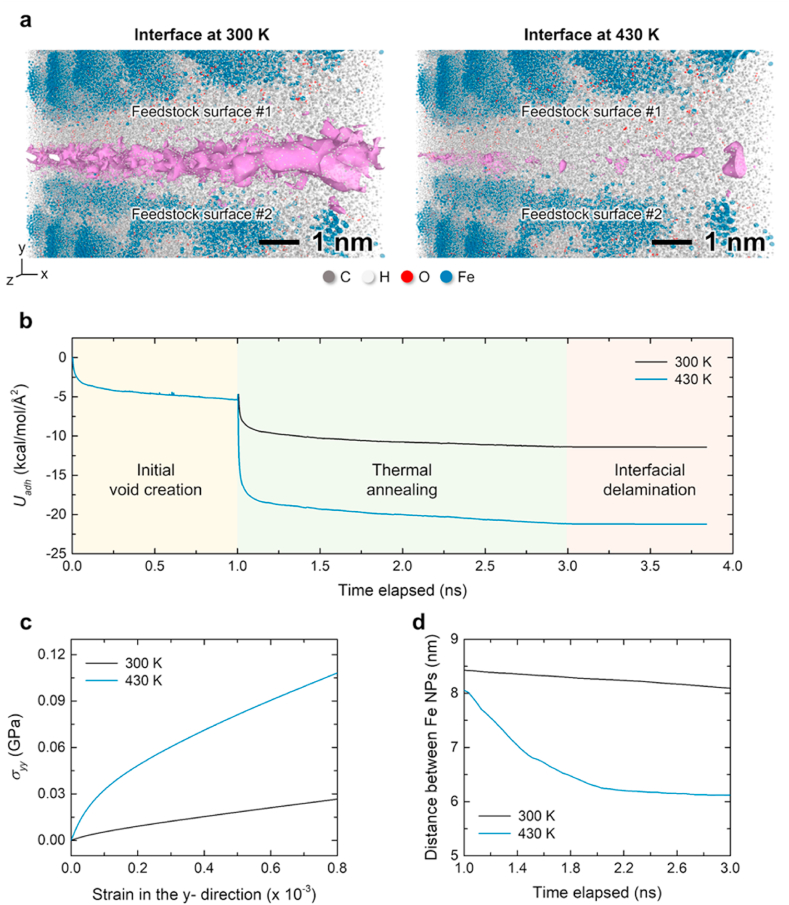
Mechanism for improving adhesion between feedstock surfaces through heat treatment. (a) Molecular structure of feedstock surfaces before and after the temperature increase, where nanoscale voids are shown at the interface (pink). (b) Changes in adhesion energy during void creation (i), thermal annealing (ii), and interfacial delamination under mechanical loading (iii). (c) Adhesive stress at feedstock interface during delamination. (d) Average distance change between iron nanoparticles on adjacent feedstock surfaces. (For interpretation of the references to color in this figure legend, the reader is referred to the Web version of this article.)

All-atom MD simulations were performed to evaluate changes in the interfacial properties of the binders within the feedstock as temperature increases. Paraffin wax (PW), polyethylene (PE), ethylene-vinyl acetate (EVA), and iron nanoparticles (iron NPs) were randomly inserted into a simulation box with periodic boundary conditions applied. The interaction force of all atoms followed the PCFF (Polymer Consistency Force Field) parameters [20], and the non-covalent bonding force between metal atoms and polymer constituent atoms was derived using the six-powered Lorentz-Berthelot mixing rule. The prepared material complex units were used to model a bilayered structure to describe the binder morphology between two feedstock surfaces in contact. Under a thermodynamic ensemble, we compared the void size formed by the interfacial region of the microstructure and the spacing between NPs inserted within the bulk region at 25 °C and 155 °C conditions. The Computational Methods section of the Supplementary Data provides details regarding the modeling and calculation methods. Other details about modeling and calculation methods are provided in the Computational Methods section of the Supplementary Data. The computational results indicate that the voids between the feedstock surfaces remained large up to 25 °C, whereas their size decreased as the temperature increased to 155 °C [Fig. 5(a)]. This was caused by an increase in fluidity after the mild heat treatment of the polymer binder. The soft-deformation process yielded similar results. The effect of improving the interface properties by heat treatment can be understood quantitatively based on the magnitude of the change in adhesion energy between the two binder surfaces [Fig. 5(b)]. The improvement in the bonding energy was maintained almost consistently despite the progressive elastic deformation in the later steps. Within the strain range considered in the simulation, the maximum stress of the structure in the delamination direction of the interfacial layer increased by approximately 3.7 times after heat treatment at 155 °C [Fig. 5(c)].

Another significant advantage of the increased fluidity of the feedstock surfaces is that it reduces the distance distribution between iron nanoparticles (NPs) in the binder. Although the diameter of iron NPs considered in the MD simulation was only a few nanometers, the average distance between adjacent particles decreased by more than 20% after heat treatment at 155 °C [Fig. 5(d)]. Meanwhile, the density of the structures formed by iron atoms in the system increased (Fig. S3). Therefore, despite the sufficient dispersal of iron NPs in the feedstock, the flow of the binder material caused them to agglomerate during transport. Consequently, the density of the iron NPs increased beyond the interface. Moreover, their structural robustness was maintained at a high density after the binder was removed during the subsequent process. This indicates that heat treatment to the feedstock along with the densification process effectively reduced the inherent voids and increased the effective density of the iron particles. Therefore, applying a high temperature causes the binder to flow and hence a reduction in inherent voids, which demonstrates the significance of subpressure-driven soft deformation.

## 4 Results and discussion

### 4.1 Subpressure-driven soft deformation of three-dimensionally printed green components

**Fig. 6:**
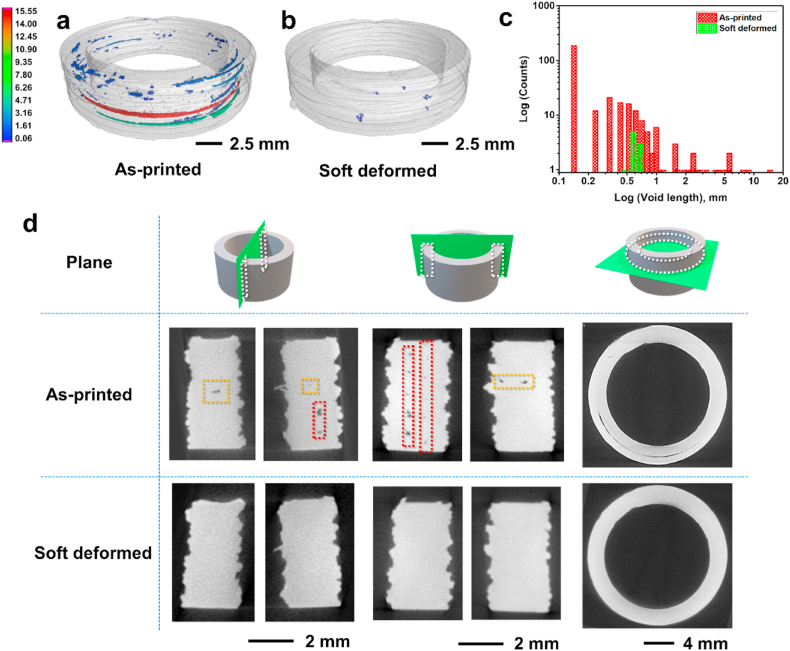
Micro-CT images showing void distribution before and after soft deformation of the same specimen. (a) As-printed specimen, (b) soft-deformed specimen (column represents void length), (c) void length distribution plot represented on a logarithmic scale, and (d) micro-CT cross-sectional images captured at the same location before and after soft deformation (dotted white boxes and circle indicate observed planes, while dotted yellow boxes indicate interlayer void, and dotted red boxes indicate intertrack voids. Finally, rightmost figure shows top view of intertrack voids). (For interpretation of the references to color in this figure legend, the reader is referred to the Web version of this article.)

Micro-CT analysis was conducted on the samples to compare the pore size distribution before and after soft deformation. Fig. 6 and S1 show the micro-CT data of the two different green components with soft deformation, which involved heat treatment at 110 °C for 15 min under subpressures of 3–4 Pa, and changes in the dimensions observed after the soft deformation are summarized in Table S1. The figures show the data before and after the soft deformation process, where significant differences in the porosity distributions of the components are indicated. In Fig. 6(a), the micro-CT data of the green components before the soft-deformation process show a wide distribution of voids. The intertrack voids, which were located between the deposited tracks and elongated along the extrusion path of the nozzle, were observed to be the longest voids and measured 5.95–15.55 mm in length (Fig. 6(a)). Another type of void, as indicated by the dotted yellow box in Fig. 6(d), was located in the middle of the deposited tracks, and its length was shorter than that of the intertrack void. This void was caused by unsatisfactory bonding with the adjacent layer during the extrusion of the three extrusion lines presented in Fig. 2(b). The third type of void is characterized by microvoids known as intra-track voids, which are round voids caused by the non-uniform distribution between the powders and binder within the deposited track during screw extrusion. This type of void is associated with surface cracks caused by stresses on the printed components during extrusion [7]. Fig. 6(b) shows the micro-CT data of the same green region after the subpressure-driven soft deformation process. As shown in Fig. 6(c), the wide distribution of pore sizes observed in the sample before soft deformation transformed into a narrow distribution, and the pore size ranged from 0.5 to 0.7 mm after soft deformation occurred. Owing to soft deformation treatment, intertrack voids with a void length exceeding 1 mm with a narrow void distribution were effectively removed, and micro-sized intra-track voids caused by stress during the extrusion process were effectively eliminated. Moreover, Fig. 6(d) shows the micro-CT data for the same sample before and after the subpressure-driven soft deformation process based on a comparison of the void distribution along various planes. The data confirmed that almost all the voids caused by unsatisfactory bonding with the subsequent layer (dotted yellow box), which are known as interlayer voids, and interlayer voids formed between the deposited tracks (dotted red box), which are known as intertracks, have been removed completely. Additionally, these two types of macrovoids transformed into micropores measuring approximately 0.7 mm through soft deformation, which was invisible along the building direction, as shown in Fig. 6(d). This show that the subpressure-driven soft deformation process effectively reduces the size of macrovoids and transforms them into a narrow distribution of smaller microvoids, thus eliminating inherent voids that cannot be removed during sintering.

### 4.2 Comparison of void distribution between green and sintered components

**Fig. 7:**
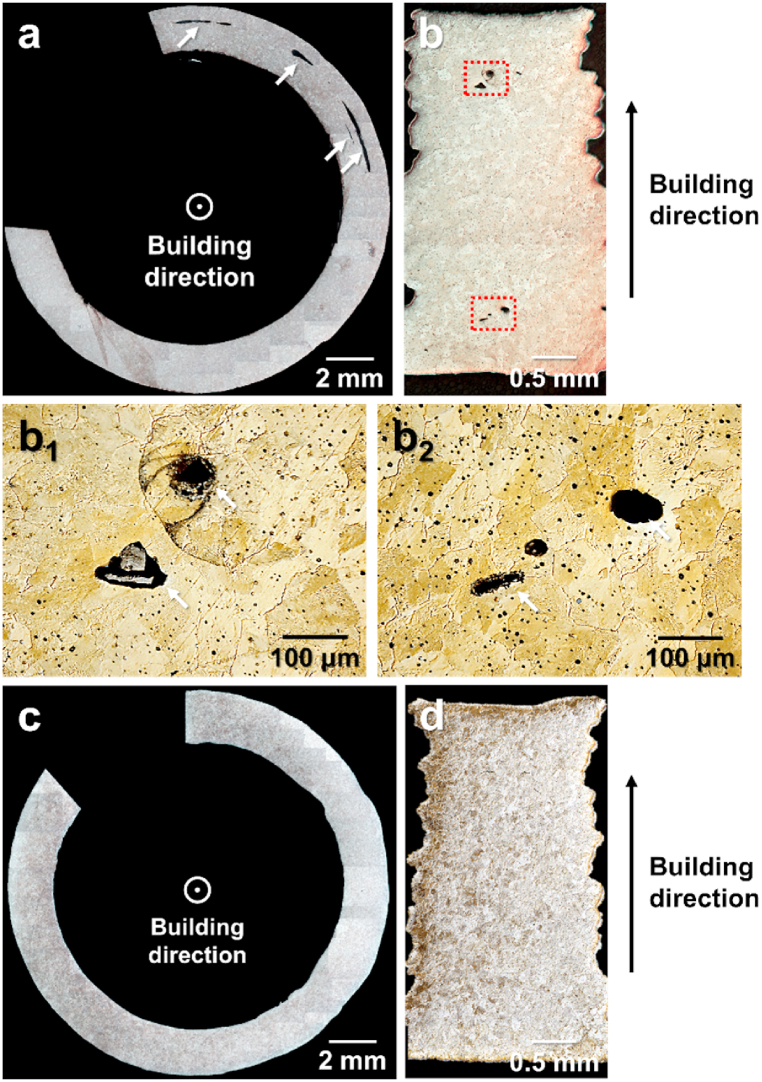
OM images of sintered component based on presence/absence of soft deformation treatment (combined from multiple images). (a) and (b) show cross-sectional images of sintered components without soft deformation; (b_1_) and (b_2_) show magnified images of area indicated by dotted red box in (b) (white arrows indicate intertrack and interlayer voids); (c) and (d) show cross-sectional images of sintered components with soft deformation. (For interpretation of the references to color in this figure legend, the reader is referred to the Web version of this article.)

A detailed investigation was performed by sintering the green components with and without soft deformation treatment and by examining the resulting microstructural differences, as shown in Fig. 7. Fig. 7 shows the cross-sectional OM images of the samples sintered in the building direction. Fig. 7(a) and (b) show the sintered samples of the as-printed green components without soft deformation. In the cross-sectional image perpendicular to the building direction in Fig. 7(a), intertrack voids, indicated by white arrows, were observed. These voids were formed between the deposited tracks (intertrack), as shown in the 3D model in Fig. 2(b). The cross-sectional images parallel to the building direction show void formation during the layer-by-layer manufacturing process of the entire specimen, where the void types can be determined from the micro-CT images (Fig. 6(d)). The images in the dotted red box in Fig. 7(b) are shown in Fig. 7(b_1_) and 7(b_2_). The voids shown in Fig. 7(b_1_), as indicated by the white arrows, were triangular intertrack voids. The void shown in Fig. 7(b_2_) was an interlayer void formed by unsatisfactory interlayer bonding. By contrast, for the samples subjected to subpressure-driven soft deformation, no significant macro-sized voids were observed in the cross-sectional images of the sintered samples, as shown in Fig. 7(c) and (d). Therefore, the voids in the green components were effectively removed through the subpressure-driven soft-deformation treatment.

**Fig. 8:**
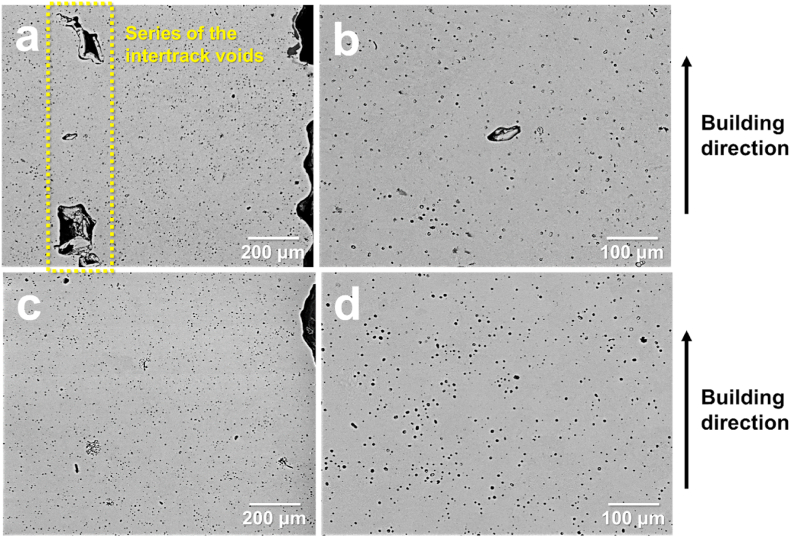
SEM images of sintered components based on presence/absence of soft deformation treatment. (a) and (b) show cross-sectional images of sintered components without soft deformation under low and high magnifications, respectively; (c) and (d) show cross-sectional images of sintered components with soft deformation under low and high magnifications, respectively.

A detailed microstructural analysis of the voids was conducted using SEM, as shown in Fig. 8. Fig. 8(a) and (b) show the sintered samples of the green components without soft deformation treatment, whereas Fig. 8(c) and (d) show the sintered samples of the green parts with soft deformation treatment. For the specimen shown in Fig. 8(a)–a series of intertrack voids was observed. This image suggests that intertrack voids cannot be easily healed during sintering. In addition to intertrack voids, micro-sized voids were observed in Fig. 8(b)–these types of voids are closed pores that appeared after the green components were sintered using the MIM feedstock system [21]. Moreover, the green components obtained in this study without high-pressure molding during MEX was more susceptible to the formation of closed pores. However, the microstructure of the green components of the sintered sample with soft deformation (Fig. 8(c) and (d)) did not exhibit a series of intertrack voids, as shown in Fig. 8(a). Fig. 8(d) shows the distribution of microsized voids in the sintered samples [similar to Fig. 8(b)]. To compare the void distributions of the sintered samples with and without soft deformation, the porosities at a minimum of four spots were analyzed. The result shows that the sintered samples from the green areas without and with soft deformation indicated average porosities of 3.55% and 2.36%, respectively. Therefore, soft deformation not only has a favorable effect on the sintering process, but also reduces the size of intertrack voids and micro-sized voids. This observation is consistent with the MD simulation results, where the average distance between adjacent particles decreased through the flow of the binder, which consequently reduced the inherent voids after heat treatment, as discussed in Section 3.2.

### 4.3 Mechanical evaluation

**Fig. 9:**
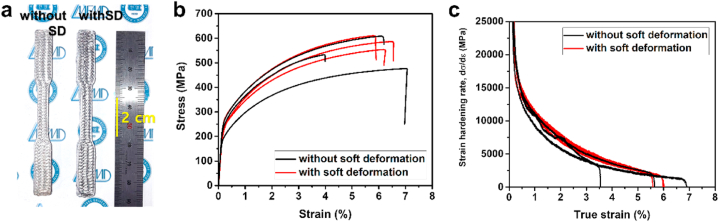
(a) Photograph of sintered components fabricated with and without soft deformation treatment on three-dimensionally printed tensile specimens. (b) Stress–strain curves of sintered samples with/without soft deformation treatment on green components. (c) Strain hardening rate curves of sintered samples with/without soft deformation treatment on green components. (For interpretation of the references to color in this figure legend, the reader is referred to the Web version of this article.)

Tensile tests were performed to evaluate the mechanical properties of the samples with and without soft deformation. To emphasize the effect of voids on the mechanical properties, the specimens were printed in a parallel–vertical–parallel–vertical (0°/90°/0°/90°) orientation with respect to the direction of tensile loading. As shown in Fig. 9(a), the feedstock was deposited perpendicular to the direction of tensile loading to fabricate the specimens for the direct evaluation of the effect of soft deformation treatment on the tensile strength. The stress–strain curves of the three sintered tensile specimens with and without soft deformation treatment are presented in Fig. 9(b)–and a summary of the corresponding data is presented in Table 2. The sintered samples subjected to the soft deformation treatment exhibited small standard deviations in their mechanical properties. However, the sintered samples without soft deformation treatment exhibited larger standard deviations in terms of the yield stress, UTS, and elongation-to-fracture than the sintered samples with soft deformation treatment. The sintered samples of the green components with soft deformation treatment showed more consistent results than those without soft deformation, possibly because of the removal of inherent voids prior to sintering. Finally, Fig. 9(c) shows the strain-hardening rate curves of the sintered samples with and without soft deformation treatment on the green components. The specimens with soft deformation treatment exhibited a consistent trend of strain hardening with similar true strain values, whereas the specimens without soft deformation exhibited a wide distribution in the true strain values, ranging from 3.53% to 6.87%.

**Table 2:** Tensile test results (three samples for each test).

	Yield stress ± dev. (MPa)	Stress at UTS ± dev. (MPa)	Strain at UTS ± dev. (%)	Elongation to fracture ± dev. (%)
without soft deformation	270.3 ± 34.6	536.3 ± 52.9	5.35 ± 1.38	5.75 ± 1.29
with soft deformation	282.1 ± 17.6	583.7 ± 22.5	5.85 ± 0.19	6.22 ± 0.28

**Fig. 10:**
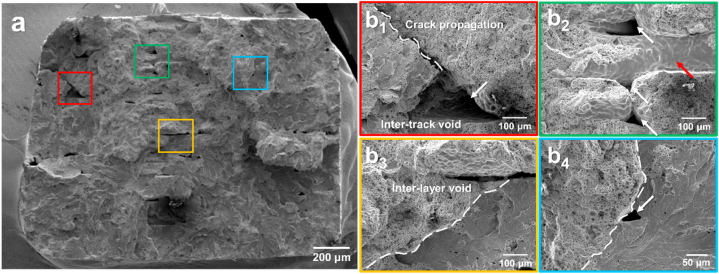
Fractography of sintered sample of green component without soft deformation. (a) shows SEM image of entire fracture surface (dashed white line indicates crack propagation), and magnified images based on each color are shown from (b_1_) to (b_4_) for (a) (white arrows indicate intertrack voids, and red arrow indicates a sintered track not bonded to the adjacent tracks). (For interpretation of the references to color in this figure legend, the reader is referred to the Web version of this article.)

**Fig. 11:**
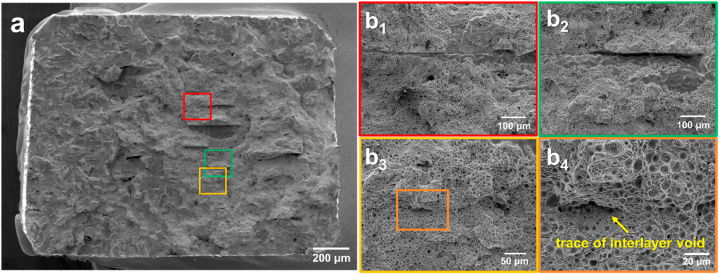
Fractography of sintered sample of green component with soft deformation. (a) shows SEM image of entire fracture surface; magnified images based on each color are shown from (b_1_) to (b_4_). (For interpretation of the references to color in this figure legend, the reader is referred to the Web version of this article.)

To further investigate the differences in the fracture behavior of the samples with and without soft deformation, the fractured surface after the tensile test was investigated, as shown in Fig. 10. Fig. 10 shows the fracture surface of the green component of the sintered sample without soft deformation. High-magnification images of the boxes shown in Fig. 10(a) are presented in Fig. 10(b_1_)–10(b_4_). Crack propagation was observed beginning from the intertrack and interlayer voids, as indicated by the dashed white lines in Fig. 10(b_1_) and 10(b_3_). As shown in Fig. 10(b_2_), the interlayer voids between the deposited tracks, which could have been removed via soft deformation, remained even during sintering. Moreover, a sintered track (which was deposited in the direction vertical to the tensile direction) that was not bonded to the adjacent tracks (red arrow) remained even after sintering, thus resulting in more intertrack voids. Finally, Fig. 10(b_4_) shows an intertrack void that resulted in a transition from a dimple to a cleavage structure in the intertrack layer, which indicates the adverse effect of these voids. Additionally, this creates stress concentrations, thus allowing cracks to initiate and propagate more easily [22]. The results above support the finding that larger standard deviations in the yield stress, UTS, and elongation-to-fracture resulted in larger standard deviations in the yield stress (Fig. 9(b)). By contrast, Fig. 11 shows the fractography of the sintered sample of the green component with soft deformation. Fig. 11(a) shows ductile dimples and cleavage structures similar to those of the samples without soft deformation (Fig. 10(a)). In the magnified images shown in Fig. 11(b_1_) to 11(b_3_), no crack propagation was observed owing to voids, whereas the various voids shown in Fig. 10 resulted in crack propagation. Moreover, Fig. 11(b_4_) shows a magnified image of Fig. 11(b_3_), where the trace of the interlayer void remained visible, although no crack propagation was observed, unlike in Fig. 10(b_1_ and b_3_). Therefore, the positive effect of the soft deformation treatment on the mechanical properties of the sintered samples was demonstrated by the elimination of inherent voids in the fractured surfaces. The consistent tensile test results and strain hardening trend further support the benefits of soft deformation in enhancing the mechanical properties. In general, these findings highlight the significant advantages of soft deformation in improving the mechanical properties of sintered samples.

## 5 Conclusion

Herein, a method for effectively removing inherent voids formed along deposited tracks during MEX to improve the sintered density of final components was proposed.

1. The viscosities of the feedstock under heat treatment at subpressures were calculated using the pressure coefficient values obtained from capillary rheometer measurement results. The viscosities were evaluated by applying a temperature higher than the melting point of the binder within the feedstock as an evaluation temperature. Viscosities ranging from 450.34 to 1018.31 Pa s were obtained under subpressure application.
2. Results of micro-CT analysis for comparing the sintered samples with and without soft deformation showed that the subpressure-driven molten feedstock removed internal voids more effectively than the feedstock without soft deformation. The soft deformation treatment effectively removed not only intertrack voids with lengths exceeding 1 mm, but also microsized intra-track voids formed by stress during the extrusion process.
3. All-atom MD simulation results confirmed that the molten feedstock effectively removed inherent voids generated between the deposited tracks, thereby improving the maximum stress on the structure in the delamination direction of the interfacial layer by 3.7 times. Moreover, the simulation results indicated that the average distance between adjacent particles decreased by more than 20%, which resulted in a more favorable arrangement for sintering.
4. The sintered sample of the green component with soft deformation showed a significant reduction in intertrack and micro-sized voids compared with the component without soft deformation. The sintered samples of the green component without and with soft deformation showed average porosities of 3.55% and 2.36%, respectively, thus indicating the favorable effect of soft deformation on the sintering process. This result is consistent with the MD simulation results, where the soft deformation treatment resulted in the rearrangement of particles to a more favorable and uniform arrangement for sintering, which was similar to the effect of particle rearrangement in the initial stage of liquid-phase sintering.
5. A comparison of the tensile strengths and fracture surfaces of the sintered samples with and without soft deformation treatment indicated that the sintered samples without soft deformation treatment exhibited larger standard deviations in terms of the yield stress, UTS, and elongation-to-fracture, whereas a narrow standard deviation was obtained for the various mechanical properties of the sintered samples with soft deformation treatment. The fractured surfaces of the sintered samples of the green component with soft deformation showed no crack propagation owing to the removal of inherent voids in the green component, whereas the large voids shown on the fracture surfaces of the sintered samples without soft deformation resulted in extensive crack propagation.

Based on our findings, subpressure-driven soft deformation is a promising solution for removing inherent voids by controlling the viscous characteristics of binders in the feedstock. This method can be applied to three-dimensionally printed components between printed lines and to all components fabricated using viscous binders to remove inevitably formed voids.

## Data availability

Data will be made available on request.

## CRediT authorship contribution statement

**Taehyeob Im:** Writing – review & editing, Writing – original draft, Visualization, Investigation, Formal analysis, Data curation, Conceptualization. **Heungseok Oh:** Validation, Investigation, Formal analysis. **Byeonghwa Goh:** Writing – original draft, Visualization, Formal analysis. **Juyong Kim:** Software, Resources. **Jai-Sung Lee:** Project administration, Methodology, Data curation, Conceptualization. **Joonmyung Choi:** Writing – review & editing, Supervision, Software, Funding acquisition, Conceptualization. **Caroline Sunyong Lee:** Writing – review & editing, Writing – original draft, Visualization, Validation, Supervision, Funding acquisition, Data curation.

## Declaration of competing interest

The authors declare that they have no known competing financial interests or personal relationships that could have appeared to influence the work reported in this paper.
