# Supplementary material for: Introduction to subpressure-driven soft deformation method for removing inherent voids in green components manufactured by material extrusion
Source: Heliyon. 2024 Mar 28;10(7):e28689. doi: 10.1016/j.heliyon.2024.e28689 (PMC11002578; doi:10.1016/j.heliyon.2024.e28689)
Supplement: Multimedia component 1 [file mmc1.docx]

**[Supplementary data]**

Introduction to Subpressure-Driven Soft Deformation Method for Removing Inherent Voids in Green Components Manufactured by Material extrusion

Taehyeob Im^1^, Huengseok Oh^1^, Byeonghwa Goh^2, 3^_,_ Juyong Kim^4^, Jai-Sung Lee^1^, Joonmyung Choi^2, 3, †^_,_ Caroline Sunyong Lee^1, †^

1. Department of Materials and Chemical Engineering, Hanyang University ERICA, Republic of Korea
2. Department of Mechanical Design and Engineering, Hanyang University, 222 Wangsimni-ro, Seongdong-gu, Seoul 04763, Republic of Korea
3. Department of Mechanical Engineering, BK21 FOUR ERICA-ACE Center, Hanyang University, 55 Hanyangdaehak-ro, Sangnok-gu, Ansan 15588, Republic of Korea
4. Reprotech 3DP R&D center, Suwon-Si, Republic of Korea

Corresponding author: (joonchoi@hanyang.ac.kr^†^), Tel: +82-31-400-5243, (sunyonglee@hanyang.ac.kr^†^), Tel: +82-31-400-4697, Fax: +82-31-263-4742

1. **Subpressure-driven soft deformation of the 3D-printed green parts**

In the micro-CT image of Figure S1(a), the other two types of voids are more prominent than the voids formed along the deposited tracks. However, it is observed that these voids have been effectively reduced in size in Figure S1(b), after undergoing the subpressure-driven soft deformation process. The detailed difference in void length is shown in Figure S1(c). As with the void length distribution presented in Figure 5(c), both voids longer than 1 mm and micro-sized pores were eliminated. As a result, the void length distribution became narrow. In Figure S1(d), numerous voids are identified by yellow dotted boxes, which are caused by weak interlayer bonding. In addition, the voids formed along the deposited tracks, identified by the red dotted box, were also eliminated after the soft deformation process. Overall, the results indicate that subpressure-driven soft deformation processe have the potential to greatly enhance the quality of green components.


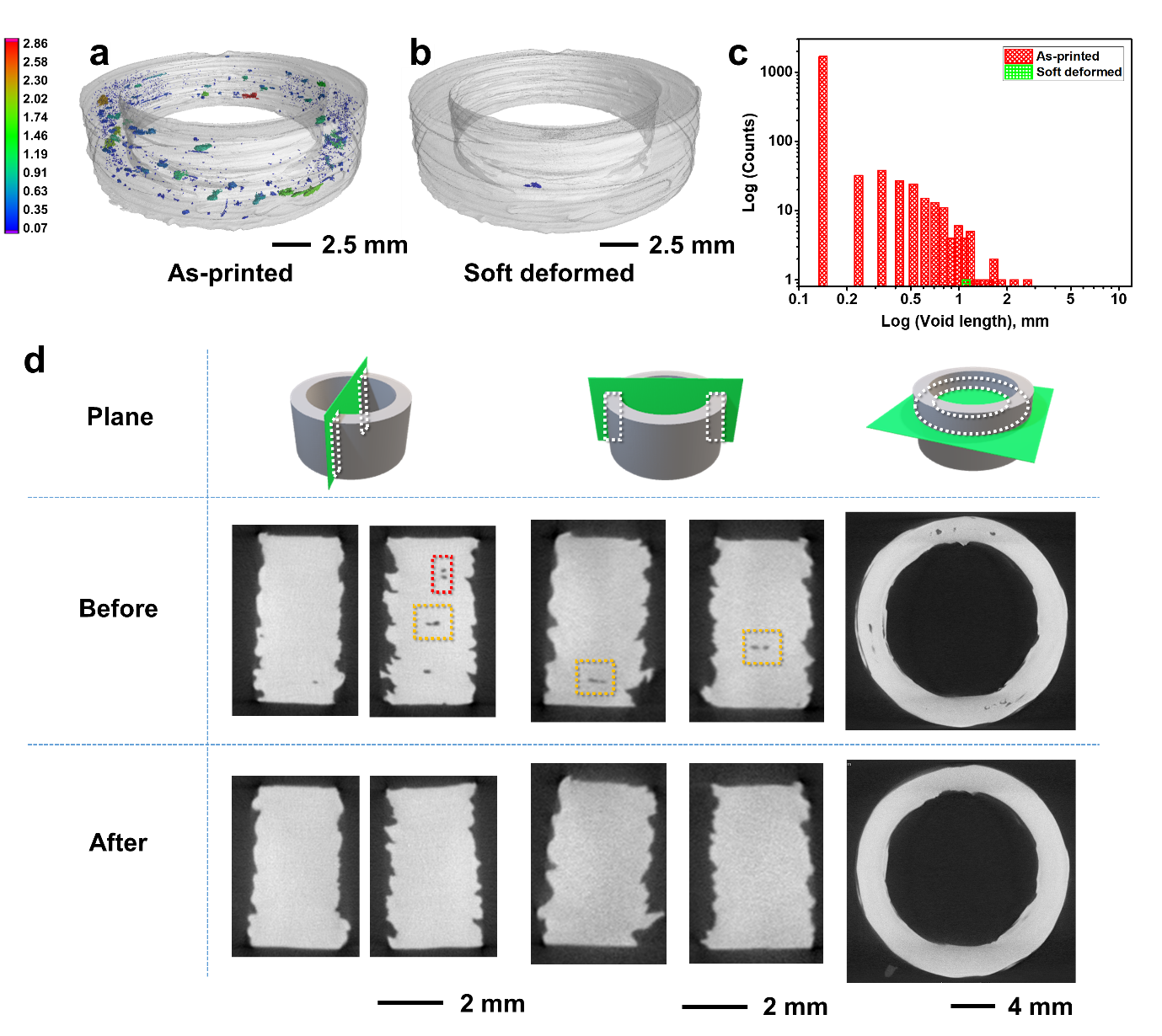


**Figure S1.** Micro-CT images showing the void distribution before and after soft deformation of the same specimen. (a) As-printed specimen, (b) Soft-deformed specimen (the column represents the void length). (c) Void length distribution plot represented on a logarithmic scale. (d) Micro-CT cross-sectional images captured at the same location before and after soft deformation (white dotted boxes and circle indicate observed planes while the yellow dotted box indicates the inter-layer void, and the red dotted boxes indicate the inter-track voids.).

**Table S1.** Design of experiments showing dimensional changes before and after the subpressure-driven soft deformation with varying soft deformation temperature and time.

| Soft deform. experiments | Conditions for  soft deform. | | Thickness (O.D.-I.D.)* | |
| --- | --- | --- | --- | --- |
|  | Temp. (℃) | Times (min.) | No soft deform. (mm) | Soft deform. (mm) |
| #1 | 110 | 5 | 2.54 | 2.50 |
| #2 | 110 | 8 | 2.59 | 2.58 |
| #3 | 110 | 10 | 2.60 | 2.61 |
| #4 | 110 | 15 | 2.55 | 2.54 |
| #5 | 110 | 18 | 2.58 | 2.94 (melt down) |
| #6 | 115 | 5 | 2.62 | 2.58 |
| #7 | 115 | 8 | 2.57 | 2.55 |
| #8 | 115 | 10 | 2.58 | 2.56 |
| #9 | 120 | 5 | 2.58 | 2.58 |
| #10 | 120 | 8 | 2.56 | 2.55 |
| #11 | 120 | 10 | 2.55 | 3.25 (melt down) |
| #12 | 125 | 5 | 2.58 | 2.59 |

* O.D.: Outer diamter, I.D.: Inner diameter

1. **Computational methods**
   1. **Molecular modeling of junctions between deposited tracks**

A periodic simulation cell box (4.62 nm × 4.62 nm × 4.62 nm) was prepared. The simulation box contained 100 paraffin wax (PW, C_30_H_62_) molecules, two polyethylene (PE, C_1000_H_2002_) molecules, two ethylene-vinyl acetate (EVA, C_1154_H_2166_O_144_) molecules, and three 2.3 nm diameter body-centered cubic iron nanoparticles (iron NPs). The initial density of the simulation box was set to 5.044 g/cm^3^, close to the density of the experimentally fabricated feedstock binder (5.0432 g/cm^3^). The polymer consistent force field (PCFF) [30] potential was used to describe the interatomic interactions. Specifically, the 9-6 Lennard-Jones (LJ) non-bonded potential function was applied to describe the interactions between iron atoms and between iron and polymer binder constituent atoms. The 9-6 LJ potential function calculation formula is as follows.

|  | $V_{LJ}=4\epsilon_{ij}\left[ \left( \frac{r_{eq}}{r} \right)^{9}-\left( \frac{r_{eq}}{r} \right)^{6} \right],$ | **(1)** |
| --- | --- | --- |

where $V_{LJ}$ is the non-bonded interaction potential and $\epsilon_{ij}$ is the potential well depth when the positions of the interpair atoms $i$ and $j$ are at an equilibrium distance of $r_{eq}$. The potential parameters between the different types of atoms (i.e., the Fe atom of nanoparticle and the C, H, and O atoms of polymers) were calculated using the six-powered Lorentz-Berthelot mixing rule, which is expressed as follows:

|  | $\epsilon_{ij}=\frac{2\sqrt{\epsilon_{i}\epsilon_{j}}r_{eq,i}^{3}r_{eq,j}^{3}}{r_{eq,i}^{6}+r_{eq,j}^{6}},$ | **(2)** |
| --- | --- | --- |
|  | $r_{eq,ij}=\left( \frac{1}{2}\left( r_{eq,i}^{6}+r_{eq,j}^{6} \right) \right)^{\frac{1}{6}},$ | **(3)** |

Also note that the LJ non-bonded interaction based on the mixing rule has been widely used to describe the structural behavior of nanocomposites composed of metal nanoparticle and polymer chains [S1, S2]. The specific values of the LJ force field parameters calculated by the mixing rule are shown in Table S2.

Table S2. Non-bonded potential parameter set used between the metal nanoparticle and the polymer constituent [30].

| Atom description | van der Waals equilibrium distance (Å) | van der Waals potential well depth (kcal mol^-1^) |
| --- | --- | --- |
| Fe of metal nanoparticle | 2.6595 | 13.8892 |
| C bonded with one H atom and two C or N atoms | 4.0100 | 0.0540 |
| C bonded with two H atoms and one C or N atom | 4.0100 | 0.0540 |
| C bonded with three H atoms | 4.0100 | 0.0540 |
| Carbonyl C of ester | 3.8100 | 0.1200 |
| H bonded to C atoms | 2.9950 | 0.0200 |
| O in carbonyl group | 3.3000 | 0.2670 |
| Ester O | 3.4200 | 0.2400 |

The potential energy of the model was minimized by the conjugate gradient method using the 0.0005 kcal/mol·Å criterion. The modeled cell box was replicated five times in the x- and y-directions. A 4 nm thick vacuum layer was added in the y-direction to account for the binder morphology between the two feedstock surfaces in contact. The void distribution structure in the interfacial region formed in a vacuum environment was secured by minimizing the potential energy between atoms through molecular mechanical simulations. An isothermal-isobaric (NPT) ensemble at -273.14 ℃ and 1 atm conditions was applied for 1 ns such that bonding between binder molecules could occur naturally inside the cell box while excluding thermal effects. The size of the cell box containing the interfacial void layer was converged to 23.1 nm × 25.9 nm × 4.66 nm (Figure S2). The atomic configuration of the polymer chains was modeled using the commercial MD software, Materials Studio^®^. Time integration was performed using the Verlet algorithm with a large-scale atomic/molecular massively parallel simulator (LAMMPS) [S3]. The surface area occupied by the voids was calculated using the Connolly surface method [S4] embedded in the open visualization tool (OVITO) [S5].

- 1. **Evaluation of changes in interfacial voids according to temperature**

Structural changes in the interfacial region were observed when temperatures of 25 ℃ and 155 ℃ were applied to the model, in which the potential energy converged completely. The two temperature conditions represent the state before and after heat treatment of the feedstock in the experiment. The MD simulation model was thermally annealed under a canonical (NVT) dynamic ensemble at 25 ℃ and 155 ℃ for 2 ns. The change in the void distribution according to the temperature application was quantified through the adhesion energy per unit area ($U_{adh}$) formed at the interface, calculated as follows [S6]:

|  | $U_{adh}=\frac{E_{sys}-E_{1}-E_{2}}{A},$ | **(4)** |
| --- | --- | --- |

where $A$ is the area of the x-z plane of the cell box, $E_{sys}$ is the total energy, and $E_{1}$ and $E_{2}$ are the independent energies of the surface components of the two feedstocks in contact.

- 1. **Mechanical tests in the direction in which interfacial delamination occurs**

The molecular structures annealed at different temperatures were uniaxially stretched in the y-direction at a strain rate of 10^-7^ /ps until the strain reached 0.0008. The purpose of this test was to clarify the structural robustness of the interfacial structure formed at each temperature. The virial stress tensor ($\boldsymbol{\sigma}$) exerted on the simulation cell during delamination is calculated as follows [S7]:

|  | $\boldsymbol{\sigma}=\frac{1}{V}\sum\left( \boldsymbol{\sigma}_{K}+\boldsymbol{\sigma}_{P} \right)=\frac{1}{V}\left[ -\sum_{i}^{N} m_{i}\left( \boldsymbol{v}_{i}\cdot\boldsymbol{v}_{i} \right)+\frac{1}{2}\sum_{i}^{N} \sum_{j\neq i}^{N} \left( \boldsymbol{r}_{ij}\cdot\boldsymbol{F}_{ij} \right) \right],$ | **(5)** |  |
| --- | --- | --- | --- |

where $V$ is the simulation cell volume, the $K$ and $P$ subscripts denote the kinetic and potential components, respectively, $m_{i}$ and $\boldsymbol{v}_{i}$ are the mass and velocity of the atom $i$, and $\boldsymbol{F}_{ij}$ is the interaction of the relative distance ($\boldsymbol{r}_{ij}$) between atoms $i$ and $j$. The delamination process simulation was performed at -273.05 ℃ under the NVT dynamic ensemble such that the kinetic term of **Eq. (2)** is negligible. To the best of the authors’ knowledge, this approach is the clearest and most common method for analyzing the mechanical behavior of atomic systems. It has been widely used by many researchers [7,8], including this study’s research group [S10-S13].

- 1. **Distance distribution between clusters of iron metal atoms**

The distribution of the iron atoms embedded in the binder was characterized by the radial distribution function (RDF,$g(R)$) whenever a stabilized structure of the feedstock interface was secured for each step. The RDF of the Fe atoms and their clusters were calculated as follows:

|  | $g\left( R \right)=\frac{dn_{R}}{4\rho\pi R^{2}dR},$ | **(6)** |
| --- | --- | --- |

where $R$ is the distance between the iron atoms, $\rho$ is the experimental density of the iron powder (7.795 g/cm^3^), and $dn_{R}$ is the number of atoms counted within the spherical shell of thickness of $dr$. The *g(R)* results for sufficiently large *R* values (i.e., conditions far exceeding the interatomic distance of iron atoms in the body-centered cubic structure) indicate the degree of aggregation between the iron particles. In the equilibrated model, the adjacent distances of iron NPs varied from 0.4 nm to 1.6 nm (not shown in the figure). Therefore, the maximum value of $R$ was set to 10 Å, allowing the detection of both interatomic and interparticle distance changes in a single RDF. The analysis was performed for the cases where only potential energy minimization was performed without temperature application and the 25 ℃ and 155 ℃ temperatures were reached. The RDF results were normalized using the population density function.

**
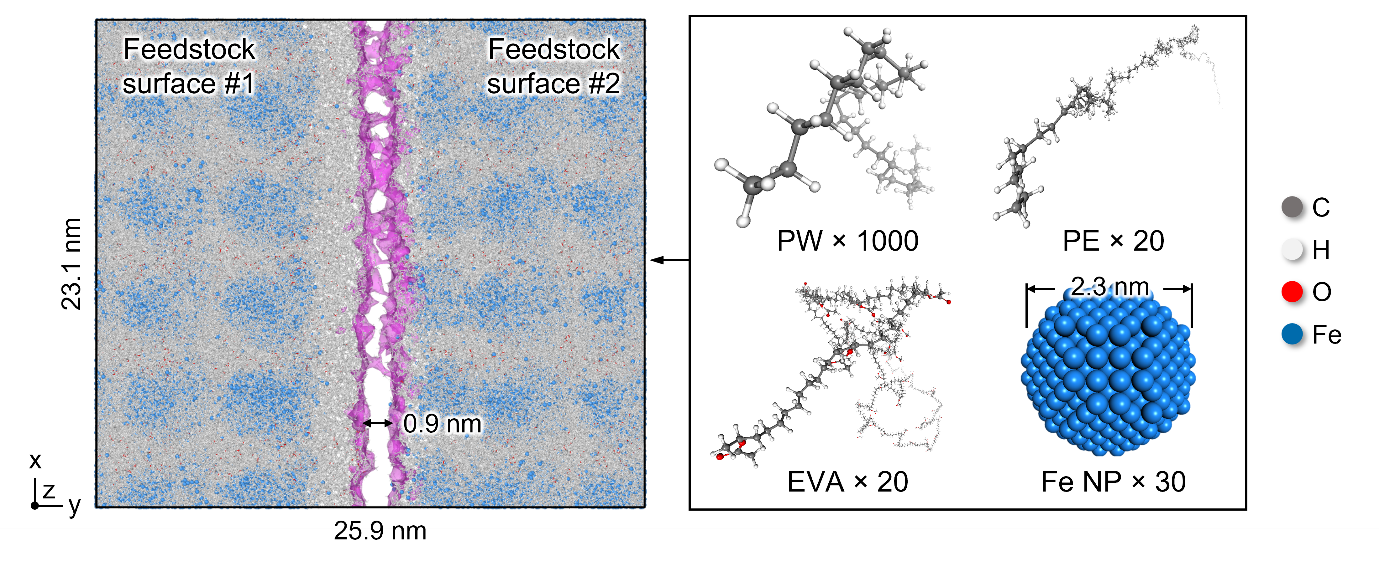
**

**Figure S2.** MD simulation model with two feedstock surfaces facing each other. The model configuration was obtained after equilibration under the NPT ensemble at room temperature and atmospheric pressure. The area occupied by the nanoscale voids at the interface is shown in pink. Molecular structures of paraffin wax (PW), polyethylene (PE), ethylene-vinyl acetate (EVA), and iron nanoparticle (iron NP) embedded in the simulation model are shown in the right panel.


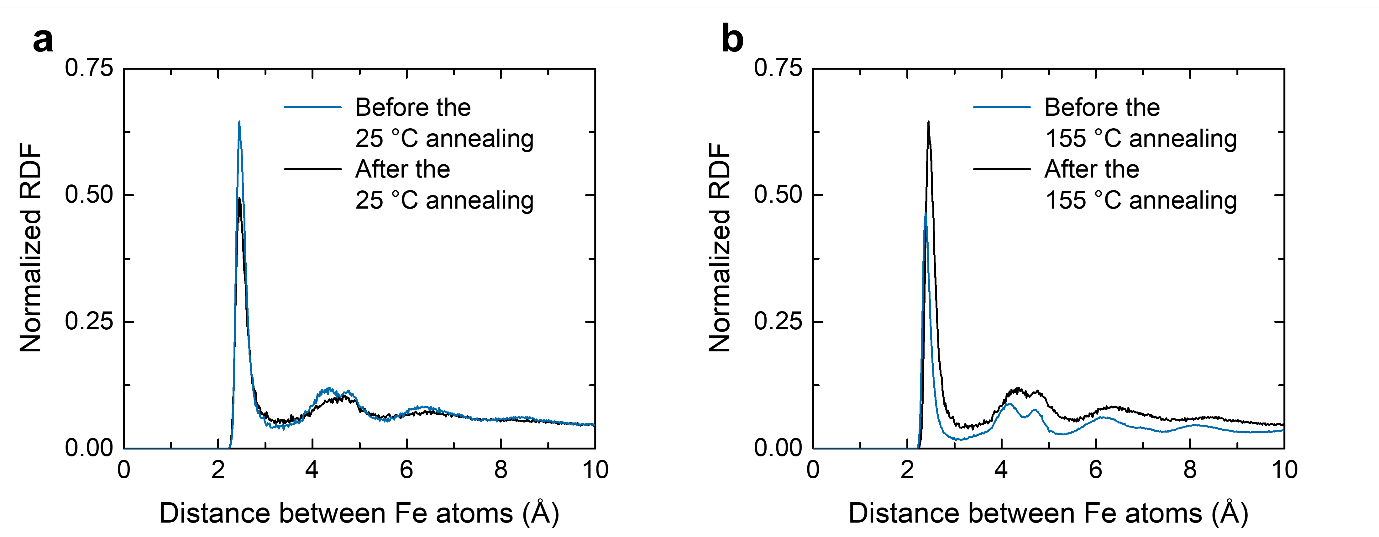


**Figure S3.** Changes in the RDF profile of iron atoms when (a) a temperature of 25 ℃ was applied after potential energy minimization and **(b)** when the temperature was increased to 155 ℃ after equilibration at 25 ℃. There is no significant difference in the distribution of peaks between the potential energy minimization condition and the 25 ℃ condition. However, for the 155 ℃ condition, it was observed that all peaks in the observation region shifted to the left, suggesting densification of iron atoms.

**Supplementary References**

[S1] J. Choi, H. Shin, M. Cho, Multiscale multiphysical analysis of photo-mechanical properties of interphase in light-responsive polymer nanocomposites, Composites Science and Technology 160 (2018) 32-41.

[S2] K. Kanhaiya, S. Kim, W. Im, H. Heinz, Accurate simulation of surfaces and interfaces of ten FCC metals and steel using Lennard–Jones potentials, npj Computational Materials 7 (2021) 17.

[S3] A.P. Thompson, H.M. Aktulga, R. Berger, D.S. Bolintineanu, W.M. Brown, P.S. Crozier, P.J. in't Veld, A. Kohlmeyer, S.G. Moore, T.D. Nguyen, LAMMPS-a flexible simulation tool for particle-based materials modeling at the atomic, meso, and continuum scales, Computer Physics Communications 271 (2022) 108171.

[S4] M.L. Connolly, Solvent-accessible surfaces of proteins and nucleic acids, Science 221(4612) (1983) 709-713.

[S5] A. Stukowski, Visualization and analysis of atomistic simulation data with OVITO–the Open Visualization Tool, Modelling and simulation in materials science and engineering 18(1) (2009) 015012.

[S6] S. Yang, J. Choi, M. Cho, Intrinsic defect-induced tailoring of interfacial shear strength in CNT/polymer nanocomposites, Composite Structures 127 (2015) 108-119.

[S7] D.M. Heyes, Pressure tensor of partial-charge and point-dipole lattices with bulk and surface geometries, Physical Review B 49(2) (1994) 755.

[S8] A.K. Subramaniyan, C. Sun, Continuum interpretation of virial stress in molecular simulations, International Journal of Solids and Structures 45(14-15) (2008) 4340-4346.

[S9] J. Choi, H. Shin, S. Yang, M. Cho, The influence of nanoparticle size on the mechanical properties of polymer nanocomposites and the associated interphase region: A multiscale approach, Composite Structures 119 (2015) 365-376.

[S10] B. Goh, K.J. Kim, C.-L. Park, E.S. Kim, S.H. Kim, J. Choi, In-plane thermal conductivity of multi-walled carbon nanotube yarns under mechanical loading, Carbon 184 (2021) 452-462.

[S11] B. Goh, J. Choi, A spatial upscaling method for describing the three-body potential of a diamond lattice structure, Applied Mathematical Modelling 108 (2022) 502-511.

[S12] H. Kim, H. Kim, J. Choi, Interface mechanics of liquid crystal polymer nanocomposites with high concentrations of MWCNTs, Composites Science and Technology 222 (2022) 109376.

[S13] Y. Kim, J. Kim, J.-W. Han, J. Choi, Multiscale mechanics of yttria film formation during plasma spray coating, Applied Surface Science 572 (2022) 151416.

This manuscript including supplementary data was proofread by 2-3 native editors who are also professionals in engineering field for language and structural edit (<https://www.editage.com/services/english-editing/premium-editing-plan>).
